# Polysomnographic phenotyping of obstructive sleep apnea and its implications in mortality in Korea

**DOI:** 10.1038/s41598-020-70039-5

**Published:** 2020-08-06

**Authors:** Jeong-Whun Kim, Tae-Bin Won, Chae-Seo Rhee, Young Mi Park, In-Young Yoon, Sung-Woo Cho

**Affiliations:** 1 Department of Otorhinolaryngology-Head and Neck Surgery, Seoul National University Bundang Hospital, Seoul National University College of Medicine, 82 Gumi-ro 173th street, Bundang-gu, Seongnam, Gyeonggi-do 13620 South Korea; 2 Department of Otorhinolaryngology Head and Neck Surgery, Seoul National University Hospital, Seoul National University College of Medicine, Seoul, South Korea; 3 Medical Research Collaborating Center, Seoul National University Bundang Hospital, Seongnam, South Korea; 4 Department of Psychiatry, Seoul National University Bundang Hospital, Seoul National University College of Medicine, Seongnam, South Korea

**Keywords:** Diseases, Medical research

## Abstract

Conventionally, apnea–hypopnea index (AHI) is used to define and categorize the severity of obstructive sleep apnea. However, routine polysomnography (PSG) includes multiple parameters for assessing the severity of obstructive sleep apnea. The goal of this study is to identify and categorize obstructive sleep apnea phenotypes using unsupervised learning methods from routine PSG data. We identified four clusters from 4,603 patients by using 29 PSG variable and arranged according to their mean AHI. Cluster 1, spontaneous arousal (mean AHI = 8.52/h); cluster 2, poor sleep and periodic limb movements (mean AHI = 12.16/h); cluster 3, hypopnea (mean AHI = 38.60/h); and cluster 4, hypoxia (mean AHI = 69.66/h). Conventional obstructive sleep apnea classification based on apnea–hypopnea index severity showed no significant difference in cardiovascular or cerebrovascular mortality (Log rank P = 0.331), while 4 clusters showed an overall significant difference (Log rank P = 0.009). The risk of cardiovascular or cerebrovascular mortality was significantly increased in cluster 2 (hazard ratio = 6.460, 95% confidence interval 1.734–24.073) and cluster 4 (hazard ratio = 4.844, 95% confidence interval 1.300–18.047) compared to cluster 1, which demonstrated the lowest mortality. After adjustment for age, sex, body mass index, and underlying medical condition, only cluster 4 showed significantly increased risk of mortality compared to cluster 1 (hazard ratio = 7.580, 95% confidence interval 2.104–34.620). Phenotyping based on numerous PSG parameters gives additional information on patients’ risk evaluation. Physicians should be aware of PSG features for further understanding the pathophysiology and personalized treatment.

## Introduction

Obstructive sleep apnea (OSA) refers to a chronic condition characterized by recurrent episodes of upper airway obstruction during sleep, leading to intermittent hypoxia, sympathetic nervous system activation, daytime sleepiness, and impaired cognitive function. OSA also has a significant impact on cardiovascular and cerebrovascular function^1^.

OSA diagnosis is typically based on in-laboratory full-night polysomnography (PSG), which collects multiple types of physiological data during sleep. Currently, the apnea–hypopnea index (AHI) is used to define and categorize the severity of OSA^2^. AHI is the most commonly reported statistically significant predictor of cardiovascular morality and all-cause mortality^3,4^. In PSG, other parameters besides AHI, such as oxygen desaturation, sleep architecture, position statistics, and periodic leg movements are reported. Moreover, these parameters are known to be independently related to disease outcome^5–8^.

In general, given the likelihood of associations between variables, patients with more severe OSA may have more severe degrees of hypoxia or sleep fragmentation^9,10^ However, the traits of OSA are complex,some patients may show no association between variables, reducing the overall probability of association between parameters. In this regard, cluster analysis using PSG variables may help to identify associations between variables and unique phenotypes. There several reports regarding cluster analysis to identify multiple phenotypes in OSA in Western countries^11–13^, however, not many studies had been published so far regarding only Asians. Therefore this study aimed to understand the characteristics of patients with OSA in Korea by identifying patient phenotypes based on multiple PSG parameters using unsupervised learning clustering methods. We also attempted to evaluate differences in the clinical outcomes of cardiovascular or cerebrovascular disease-related mortalities or all-cause mortality among the different phenotypes.

## Methods

### Study population

This was a single tertiary center study based on a retrospective review of medical records. Our study population consisted of patients who had visited the tertiary sleep center for snoring or sleep apnea from January 2007 to October 2017 and underwent a full-night in-laboratory PSG. Patients below 18 years of age or those with a total sleep time less than 240 min were excluded as in our previous study^3^. Follow up PSGs taken after treatment such as surgery, application of mandibular advancement devices, or PSG taken for titration of continuous positive airway pressure (CPAP) were also excluded. After PSG variables has been selected for further analysis which will be described in the following section, patients with missing variables were excluded. Thus, the final sample size in our study cohort was 4,603 patients (67.6%) out of 6,803 patients. Among the patients, 3,324 were men and the other 1,279 were women. Mean age was 51.9 ± 14.3 years. Duration of recording (time in bed) and total sleep time ranged from 322–720 min to 240–657 min, respectively. Medical condition of enrolled patients was analyzed by their diagnosis based on International Classification of Diseases and Related Health Problems, 10th Revision (ICD‐10). Serum total cholesterol, high density lipoprotein (HDL) levels and smoking status were collected during the study period. Smoking status was reported as never smoker or current/ex-smoker. Serum total cholesterol, HDL level that had been checked were averaged for each patient. Among the patients, 3,299 (71.7%), 2,381 (51.7%), and 1,978 (42.4%) patients were evaluated for serum total cholesterol, HDL, and smoking status. Since not all patients were evaluated, these factors were not adjusted for survival analysis.

All data had been acquired by the Clinical Data Warehouse of hospital electronic medical record system (Bestcare, Ezcaretech, Seoul, Korea)^14^. As this is a retrospective study with minimal risk for patients, requirement of informed consents was waived by the institutional review board of Seoul National University Bundang Hospital. All methods were carried out in accordance with the ethical standards of the 1964 Helsinki declaration and its later amendments. This study was approved by the institutional review board (IRB No. B-1804/465-104).

### Full-night in-laboratory PSG

All subjects underwent a full-night in-laboratory PSG using an Embla N7000 recording system (Embla; Medcare, Reykjavik, Iceland) with the supervision of an experienced technician at the sleep center, as previously described^15^. PSG parameters included the following: electroencephalography, electrooculography, submental electromyography, lower leg electromyography, electrocardiography, leg movement, chest and abdominal movements, nasal airflow, mouth airflow (thermistor), pulse oximetry, and body position. Based on standard criteria, sleep stages were scored in 30-s epochs. Apnea was defined as an absence of airflow for ≥ 10 s, and were classified as obstructive, mixed, or central according to the American Academy of Sleep Medicine (AASM) recommended guidelines^16^. Hypopnea was defined as a > 50% decrease in airflow for at least 10 s or a moderate reduction in airflow for at least 10 s associated with arousals or oxygen desaturation (< 4%)^17^. AHI was calculated as the total number of apneas and hypopneas per hour sleep.

### Variable reduction analysis

A two-step variable reduction analysis was conducted. Briefly, clinically relevant PSG variables were selected, followed by principal component analysis-based dimension reductions and K-means cluster analysis^18^.

PSG results consisted of 62 variables (Supplementary Table S1). Among them, 29 PSG variables were selected (Table 1). Three practicing sleep clinicians (JW Kim, IY Yoon, and SW Cho) at the Seoul National University Bundang Hospital reviewed and selected seemingly clinically relevant variables. To identify phenotypes solely using PSG parameters, other measurements, such as age, sex, and anthropometric measurements, including body mass index (BMI) and neck circumference, were excluded. Moreover, sleep questionnaire scores, including the Epworth Sleepiness Scale (ESS) score and Pittsburg Sleep Quality Index (PSQI) score, were not used as variables. Redundant variables (eg. AHI in non-supine position) that could be easily extracted from the other variables (AHI, supine AHI, and percent sleep time in supine position) were excluded. As we considered to analyze patients whose total sleep time is more than 4 h, total sleep time, time in bed (sum of sleep latency, time of wake after sleep onset, and total sleep time) were not considered. Instead, we selected sleep efficiency which could represent these time variables. PSG variables were categorized into the domains of sleep architecture, breathing disturbance, desaturation, limb movement, and arousal, based on known mechanisms of cardiovascular consequences of OSA^4,19^ which is similar to a recent study by Zinchuk et al.^13^_._ Although most of the variables are overlapping with those from an aforementioned study, several different variables were selected. Durations of respiratory events were included for analysis since these variables are known to differ among patients with similar AHI, with different cardiovascular outcome^20,21^. The central apnea index was not included in variable reduction or cluster analysis, since among the variables in the apnea index category, the distribution of the central apnea index was the most skewed [skewness = 19.4, with D (4,603) = 0.417 and P < 0.001 by Kolmogorov–Smirnov test for normality], and therefore, was not suitable for standard principal component analysis^22^. AHI is a composite of variables that it could be easily calculated as sum of apnea index and hypopnea index. However, as AHI is the most commonly reported statistically significant predictor of cardiovascular morality and all-cause mortality, AHI was included in our study. Finally, sleep position in OSA was also considered. Therefore, AHI in supine position, time percent of supine position during sleep was considered.

**Table 1:** Polysomnographic parameters used for analysis.

Domain	Parameters
Sleep architecture	Sleep latency (min)
	Sleep efficiency (%)
	REM latency (min)
	Stage1 NREM (% of total sleep time, TST )
	Stage2 NREM (% of TST)
	Stage3 NREM (% of TST)
	Stage REM (% of TST)
Breathing disturbance	AHI (events/h)
	Apnea index (events/h)
	Obstructive apnea index (events/h)
	Mixed apnea index (events/h)
	Hypopnea index (events/h)
	AHI during REM sleep (events/h)
	AHI during NREM sleep (events/h)
	AHI in supine position (events/h)
	Supine percent (% of TST)
	Mean apnea duration (s)
	Mean hypopnea duration (s)
	Mean apnea–hypopnea duration (s)
Desaturation	Mean O_2_ saturation
	Lowest O_2_ saturation
	Time of O_2_ saturation < 90% (% of TST)
	ODI (events/h)
Limb movement	Limb movement (events/h)
	PLM (events/h)
Arousal	Limb movement with arousal (events/h)
	Respiratory arousal (events/h)
	PLM arousal (events/h)
	Spontaneous arousal (events/h)

*AHI* Apnea–hypopnea index, *NREM* non rapid eye movement, *ODI* oxygen desaturation index, *PLM* periodic limb movement, *REM* rapid eye movement.

After variables for analysis has been selected, patients with missing variables were excluded.

### Cluster analysis

Variables were standardized with a mean of zero and standard deviation of one. Using these variables as input, principal components analysis was used to derive a set of low-dimensional components while still preserving as much variance as possible. Selection of components that retained at least 75% of the total variance (the sum of variances of all individual principal components) were determined^23^ (Supplementary Table S2). After application of varimax rotation to maximize item variance and simplify interpretability, scores of given principal components representing PSG features were acquired for each subject^24^. The number of clusters from this dataset was then estimated via the gap static^25^ with 500 bootstraps. The suggested number of clusters was 4 (Supplementary Figure S1). Thereafter, K-means cluster analysis was performed using the factors. Cluster validation had been performed by calculation of Dunn index and average silhouette width index^26^ (Supplementary Table S3).

### Mortality status

Outcomes of different clusters were evaluated using mortality status. Deaths that occurred up to December 31, 2016, were identified by matching each subject’s Korean Identification Number and name with death records provided by Statistics Korea (https://kostat.go.kr). The date and primary cause of death were collected from the national death statistics. Causes of death were classified based on the underlying causes described in the deceased’s death certificate, as recommended by the World Health Organization^27^.

Disease-specific causes of death in the following categories were evaluated: (1) cardiovascular or cerebrovascular diseases, such as stroke, acute myocardial infarction, acute ischemic heart disease, sudden cardiac arrest, and cardiac dysrhythmias, (2) cancer, (3) fatal events including car accidents injuries, falls, and suicide, (4) others, including pneumonia, gastrointestinal bleeding, end-stage renal disease, amyloidosis, etc^3^.

### Statistical analysis

PSG and other quantitative clinical variables were described separately for each cluster using mean and standard deviation. Comparisons between clusters were performed using one way analysis of variance for continuous variables, and the chi-squared test for categorical variable. The relationships between clusters and both disease-specific and all-cause mortality rates were evaluated using Kaplan–Meier survival analysis. A Cox proportional hazards regression analysis was used to estimate hazard ratios (HR) and 95% confidence intervals (CI), which were adjusted for age, sex, BMI, and underlying medical condition. Cox–Snell residuals were used to evaluate the overall fitness of the model^28^. Medical condition of each patient was then grouped into three disease category; hypertension, diabetes, cardiovascular/cerebrovascular disease (such as atrial fibrillation, ischemic heart disease and stroke). A repeated analysis was performed using the different categories of AHI severity (normal, AHI < 5; mild, AHI < 15; moderate, 15 ≤ AHI < 30; and severe, AHI ≥ 30) to assess whether the risk of disease-specific and all-cause mortality can be observed by conventional OSA severity categories^29^.

Analyses were performed using SPSS 22.0 (IBM Corp., Armonk, NY, USA) and R 3.4.2 software (R Foundation for Statistical Computing; https://www.r-project.com). Values of P < 0.05 were considered statistically significant.

## Results

### General characteristics of patients

Final analysis was performed with 4,603 patients (PSGs). The mean AHI was 22.6 ± 22.3 events/h. According to current classification for OSA severity based on AHI, the number of patients in each severity category was 1,194 (25.9%), 1,081 (23.5%), 975 (21.2%), and 1,353 (29.3%) for AHI < 5 (normal), 5 ≤ AHI < 15 (mild), 15 ≤ AHI < 30 (moderate), and AHI ≥ 30 (severe), respectively. There were significant differences in age, sex, BMI, ESS, and PSQI according to current classification for OSA severity (P < 0.001). General patient characteristics are shown in Table 2

**Table 2:** Patients’ general characteristics.

	Normal	Mild	Moderate	Severe	P value
N (%)	1,194 (25.9)	1,081 (23.5)	975 (21.2)	1,353 (29.4)	
AHI (/h)	1.9 ± 1.5	9.4 ± 2.9	21.6 ± 4.2	52.1 ± 17.0	< 0.001
SE	0.4	0.9	0.13	0.46	
Range	0–4.9	1.4–14.9	15.0–29.9	30–129.3	
Age (years)	49.2 ± 16.2	53.1 ± 14.1	53.9 ± 13.4	51.8 ± 12.9	< 0.001
SE	0.47	0.43	0.43	0.35	
Range	18.0–88.3	18.3–89.5	18.5–89.8	20.7–94.0	
Sex (male)	613 (51.3%)	755 (69.8%)	771 (79.1%)	1,185 (87.6%)	< 0.001
BMI (kg/m^2^)	23.7 ± 3.1	25.0 ± 3.3	25.7 ± 3.2	27.4 ± 3.8	< 0.001
SE	0.9	0.1	0.1	0.1	
Range	15.2–36.2	13.5–40.4	14.2–40.5	17.6–51.9	
ESS	8.5 ± 4.8	9.2 ± 5.0	9.5 ± 4.9	10.7 ± 5.0	< 0.001
SE	0.15	0.16	0.16	0.14	
Range	1–31	1–27	1–24	1–29	
PSQI	9.1 ± 4.8	8.6 ± 4.5	7.8 ± 5.2	7.3 ± 3.9	< 0.001
SE	0.14	0.14	0.17	0.11	
Range	1–21	1–23	1–101	1–40	

*AHI* apnea–hypopnea index, *BMI* body mass index, *ESS* Epworth sleepiness scale, *PSQI* Pittsburg sleep quality index, *SE* standard error.

### Cluster characteristics

**Figure 1:**
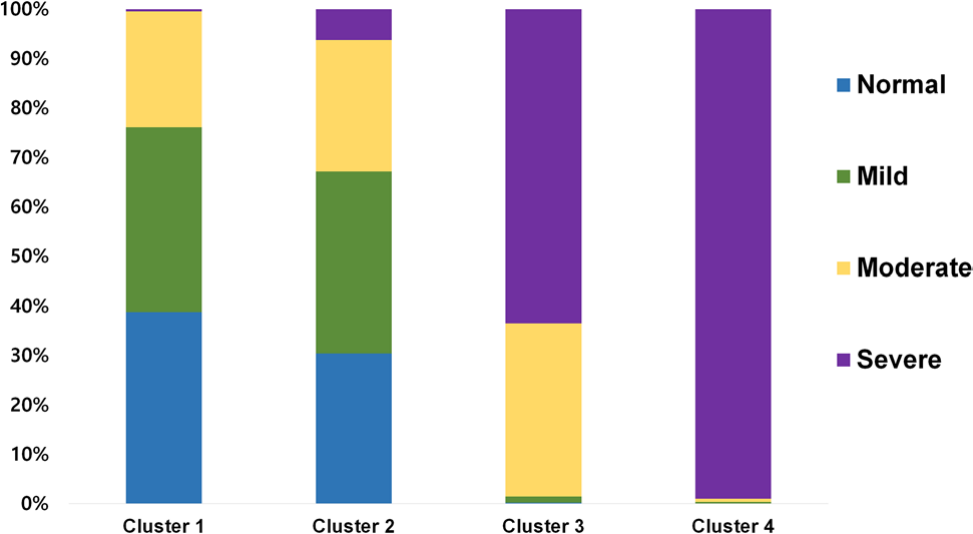
Stacked bar graph showing the distribution of OSA severity in each cluster based on AHI (normal: AHI < 5; mild: AHI < 15; moderate: 15 ≤ AHI < 30; and severe: AHI ≥ 30). *AHI* apnea–hypopnea index.

Twenty-nine PSG parameters were reduced into nine factors, and based on these factors, K-means cluster analysis resulted in four OSA subgroups. Subsequently, the four clusters were rearranged according to their mean AHI, similar to conventional categorization of OSA severity categorized based on AHI. A summary of cluster characteristics is described in Table 3. All 29 PSG variables used for analysis differed significantly among the four clusters (P < 0.001). Other clinical parameters that were not used for analysis, such as age, BMI, ESS, and PSQI, were also significantly different among the four clusters (P < 0.001). Cluster labeling was based on AHI severity and differences in PSG features between clusters. Distribution of patients’ OSA severity, according to the AHI within each cluster, is described in Fig. 1.

**Table 3:** Cluster characteristics.

	Clusters				P value*
	1	2	3	4	
N (%)	2,563 (55.7)	352 (7.6)	1,278 (27.8)	410 (8.9)	
Summary	Normal to mild OSA, spontaneous arousal	Normal to mild OSA, poor sleep, PLM	Moderate to severe OSA, hypopnea	Severe OSA, hypoxemia	
**Polysomnographic parameters**					
AHI(/h)	8.52 ± 7.43	12.16 ± 11.61	38.60 ± 13.65	69.66 ± 14.54	< 0.001
SE	0.15	0.62	0.38	0.72	
Range	0–71.5	0–68.0	0.5–102.4	12.6–129.3	
Obstructive apnea index (/h)	2.53 ± 3.50	4.66 ± 6.84	19.60 ± 11.54	51.44 ± 18.32	< 0.001
SE	0.7	0.36	0.32	0.90	
Range	0–23.1	0–59.5	0–104.0	0.2–101.60	
Hypopnea index (/h)	5.64 ± 5.32	7.03 ± 7.13	16.63 ± 10.47	9.94 ± 9.20	< 0.001
SE	0.11	0.38	0.29	0.45	
Range	0–33.7	0–52.6	0–71.4	0–52.5	
Sleep efficiency (%)	81.67 ± 10.46	74.65 ± 11.03	81.31 ± 10.49	84.41 ± 9.75	< 0.001
SE	0.21	0.59	0.29	0.48	
Range	15.0–99.2	44.7–96.8	12.0–98.5	52.9–99.0	
Stage1 NREM (% of TST)	9.27 ± 4.85	10.23 ± 5.86	15.82 ± 7.49	24.32 ± 11.54	< 0.001
SE	0.1	0.31	0.21	0.57	
Range	0.1–32.5	1.4–39.3	1.4–58.8	0.6–72.6	
Stage3 NREM (% of TST)	8.61 ± 7.57	6.03 ± 6.84	5.84 ± 5.94	3.24 ± 4.43	< 0.001
SE	0.15	0.36	0.17	0.22	
Range	0–45.7	0–36.3	0–34.5	0–24.1	
REM (%)	16.57 ± 6.63	14.51 ± 6.56	15.45 ± 6.32	14.94 ± 6.26	< 0.001
SE	0.13	0.35	0.18	0.31	
Range	0.4–54.0	1.0–51.1	0.7–43.6	1.0–36.5	
AHI REM/NREM	3.7 ± 9.02	2.63 ± 7.02	1.22 ± 1.21	0.86 ± 0.27	< 0.001
SE	0.18	0.38	0.03	0.01	
Range	0–187.0	0–81.75	0–15.73	0.02–2.85	
AHI supine/lateral	13.17 ± 21.35	14.18 ± 31.42	11.53 ± 26.30	2.85 ± 7.80	< 0.001
SE	0.57	2.09	0.81	0.44	
Range	0–201.87	0–397.4	0.45–359.67	0–118.33	
Time of O_2_ saturation < 90% (% of TST)	0.44 ± 1.36	1.35 ± 4.87	6.33 ± 7.61	30.65 ± 19.18	< 0.001
SE	0.03	0.26	0.21	0.95	
Range	0–27.9	0–70.8	0–90.8	0–100.0	
Mean apnea hypopnea duration (s)	24 ± 7.59	24.44 ± 6.65	26.79 ± 6.19	30.58 ± 7.47	< 0.001
SE	0.15	0.35	0.17	0.37	
Range	0–67.9	0–44.5	13.5–56.5	14.5–59.6	
PLM (/h)	4.33 ± 9.31	61.96 ± 33.39	3.8 ± 9.02	0.93 ± 4.05	< 0.001
SE	0.18	1.78	0.25	0.2	
Range	0–57.1	12.5–262.0	0–67.4	0–37.1	
Respiratory arousal (/h)	5.33 ± 5.35	7.78 ± 8.75	27.79 ± 12.50	60.071 ± 4.71	< 0.001
SE	0.11	0.47	0.35	0.73	
Range	0–48.2	0–64.3	0.2–89.9	6.0–118.6	
Spontaneous aousal (/h)nf	5.28 ± 4.13	3.22 ± 2.63	2.81 ± 2.05	0.79 ± 0.98	< 0.001
SE	0.08	0.14	0.06	0.05	
Range	0–89.1	0–14.5	0–15.9	0–8.5	
**Non-polysomnographic parameters**					
Age (year)	49.94 ± 14.83	63.91 ± 10.36	53.63 ± 12.97	48.18 ± 11.52	< 0.001
SE	0.29	0.55	0.36	0.57	
Range	18.01–89.81	27.68–88.0	20.68–93.96	21.67–83.37	
BMI (kg/m^2^)	24.55 ± 3.26	24.79 ± 3.32	26.59 ± 3.26	28.92 ± 4.24	< 0.001
SE	0.06	0.18	0.09	0.21	
Range	13.5–40.5	14.2–40.4	17.6–46.8	19.6–51.9	
ESS	9.08 ± 4.90	8.44 ± 4.98	10.01 ± 4.98	11.69 ± 4.86	< 0.001
SE	0.1	0.29	0.14	0.25	
Range	1–31	1–24	1–29	1–24	
PSQI	8.57 ± 4.55	9.4 ± 7.15	7.5 ± 3.92	7.02 ± 3.81	< 0.001
SE	0.09	0.41	0.11	0.19	
Range	1–27	1–101	1–20	1–40	

*AHI* Apnea–hypopnea index, *NREM* non rapid eye movement, *ODI* oxygen desaturation index, *PLM* periodic limb movement, *REM* rapid eye movement, *BMI* Body mass index, *ESS* Epworth sleepiness scale, *PSQI* Pittsburg sleep quality index.

*By one way ANOVA.

### Cluster 1

Cluster 1 was the largest among the 4 clusters (n = 2,563, 55.7%). The mean AHI was 8.52 ± 7.43/h, the lowest among all other clusters. This cluster tended to have the lowest proportion of stage 1 non-rapid eye movement (NREM) sleep, 9.27 ± 4.85% of total time analyzed, while other sleep stages were longer compared to other clusters. This cluster had the highest spontaneous arousal index (5.28 ± 4.13/h) among all clusters. This cluster was labeled as “normal to mild OSA with spontaneous arousal”.

### Cluster 2

Cluster 2 was the least common, comprising only 7.6% (352/4,603). The mean AHI was 12.16 ± 11.61/h, significantly higher than that of cluster 1 (P < 0.001). This cluster had the longest sleep latency (26.19 ± 31.42 min) and REM latency (155.99 ± 96.94 min) and the lowest sleep efficiency (74.65 ± 11.03%). The most significant PSG parameters in this cluster were periodic limb movement (PLM) index score (61.96 ± 33.34/h) and PLM associated arousal index (10.93 ± 11.58/h). This cluster was the oldest with a mean age of 63.91 ± 10.36 years. ESS score was the lowest (8.44 ± 4.98) in this cluster, while PSQI score was the highest (9.40 ± 7.15). This cluster was labeled as “normal to mild OSA with poor sleep and PLMs”.

### Cluster 3

Cluster 3 was the second largest cluster, comprising 27.8% (1,278/4,603) of the patients. The mean AHI was 38.60 ± 13.65/h. Among the analyzed PSG parameters, hypopnea index scores were the highest in this cluster (16.63 ± 10.47/h). This cluster was labeled as “moderate to severe OSA with hypopnea”.

### Cluster 4

Cluster 4 comprised 8.9% (410/4,603) of the patients and had the highest AHI with a mean value of 69.66 ± 14.54/h. This cluster had the shortest sleep latency (11.59 ± 16.22 min) and the highest sleep efficiency (84.40 ± 9.75%). Compared to other clusters, this cluster had the highest proportion of stage 1 NREM sleep (24.31 ± 11.54%) and the lowest proportion of stage 2 and 3 NREM sleep (44.09 ± 12.71%, 3.24 ± 4.43%, respectively). The oxygen desaturation index (ODI), percentage of time with SpO_2_ less than 90%, respiratory arousal index, mean apnea and hypopnea duration, were the highest in cluster 4 among all of the four clusters. Compared to all other clusters, this cluster was apnea dominant with the lowest proportion of hypopnea index over AHI (14.54 ± 13.81%). AHI during REM over AHI during NREM was the lowest in cluster 4 with a ratio of 0.86 ± 0.27. Regarding AHI according to sleep position, this cluster had the lowest ratio of supine AHI over lateral AHI (2.85 ± 7.80). Among non-PSG features, this cluster had the highest ESS score (11.69 ± 4.86), highest BMI (28.92 ± 4.24 kg/m^2^), and the youngest age (48.18 ± 14.30 years). This cluster was labeled as “severe OSA with hypoxemia”.

Obesity is an important risk factor for OSA and many PSG features show correlation with BMI^30,31^. Because the BMI of cluster 4 was the highest, the PSG features were compared for subgroup analysis according to BMI status (BMI ≥ 25 kg/m^2^, and BMI < 25 kg/m^2^) to evaluate the impact of obesity. Among 410 patients, 68 were non-obese (BMI < 25 kg/m^2^) while 342 were obese (BMI ≥ 25 kg/m^2^). The mean AHI was significantly higher in obese patients than in non-obese patients (71.02 ± 14.29 vs 62.80 ± 13.92, P < 0.001). However, there was no significant difference in AHI during REM over AHI during NREM, ratio of supine AHI over lateral AHI, and fraction of hypopnea index between the 2 groups. Interestingly, the mean total apnea–hypopnea duration was significantly higher in non-obese patients (34.39 ± 7.81 vs 29.83 ± 7.18, P < 0.001). The PSG features of obese and non-obese patients in cluster 4 are summarized in Supplementary Table S4.

### Survival analysis

**Figure 2:**
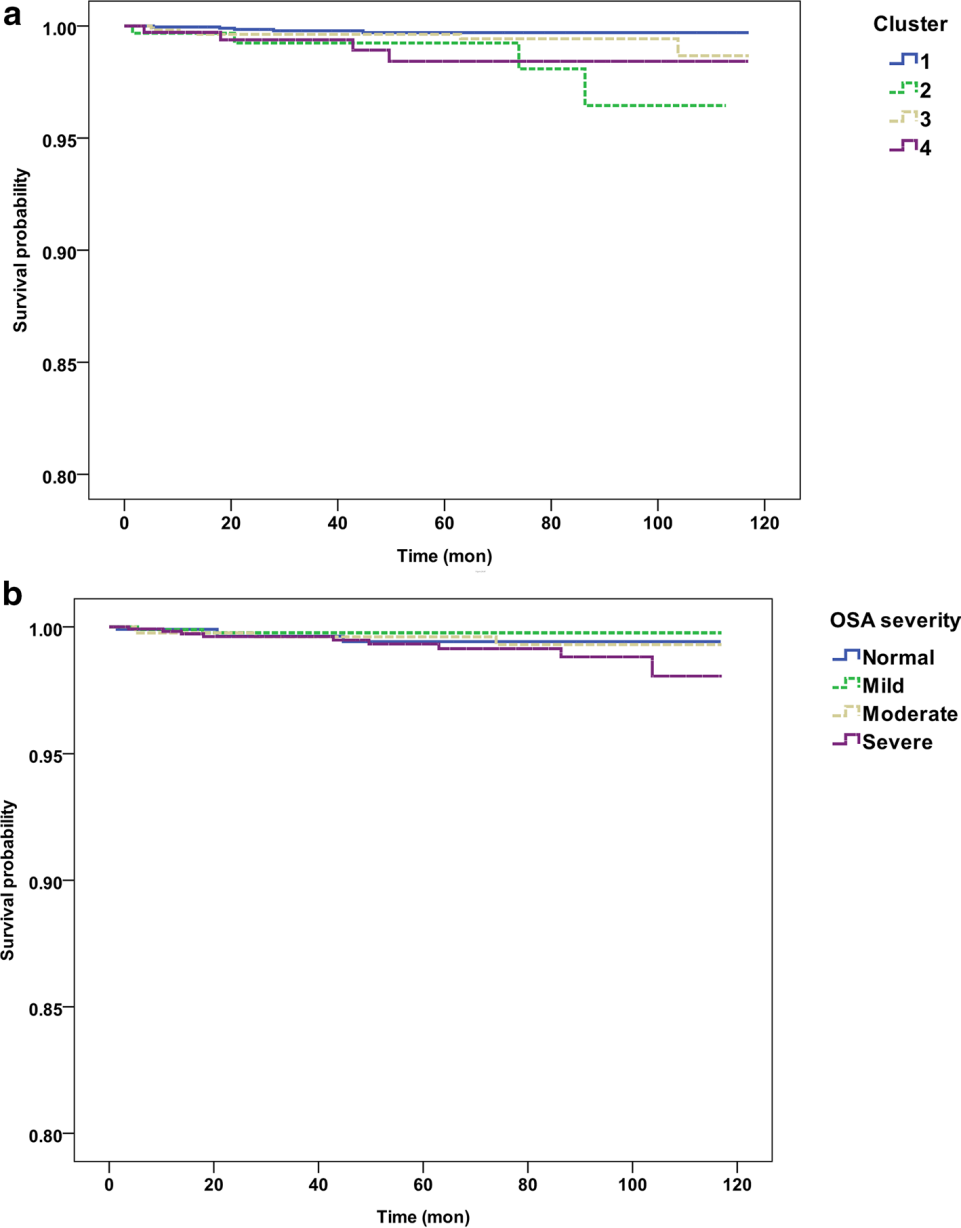
Kaplan–Meier survival probability curves for risk of cardiovascular/cerebrovascular disease specific mortality according to clusters resulted from cluster analysis (**a**) and groups of patients categorized by conventional OSA severity classification (**b**). There is a significant difference in disease specific mortality when patients are grouped by cluster analysis (Log rank P = 0.009) while as when patients are grouped by conventional OSA severity classification, there is no such significant difference (Log rank P = 0.331).

Of the 4,603 patients, survival data were acquired for 4,210 (91.5%) patients. The mean observational period was 53.49 ± 34.33 months and 119 deaths (2.82%) occurred during the observation period. Among them, 15.97% (19/119) of the deaths were due to cardiovascular or cerebrovascular disease. Other causes of death were cancer (35.2%, 42/119), fatal events (15.96%, 19/119), and others (32.8%, 39/119). The 10-year actuarial overall survival was 93.0% with an actuarial mean survival of 113.5 months (95% CI 112.8–114.1). Number of deaths and underlying medical condition, serum total cholesterol, HDL, and smoking status according to cluster are described in Table 4. Underlying comorbidities, serum cholesterol level, and smoking status were significantly different among clusters. Cluster 4 tended to have the highest prevalence of hypertension, diabetes, and smoking while having lowest HDL level. Similar table was made for OSA severity based on AHI as Supplementary Table S5. Kaplan–Meir survival analysis showed a significant difference in cardiovascular or cerebrovascular disease-specific mortality, according to clusters (Log rank, P value = 0.009, Fig. 2a). Clusters 2 and 4 showed 6.4- and 4.8-fold greater risk of mortality due to cardiovascular or cerebrovascular disease-specific mortality, respectively, compared to that in cluster 1 (Table 5). However, after adjustment for age, BMI, sex, and underlying medical condition only cluster 4 had a significantly higher risk for disease-specific mortality as compared to cluster 1 (HR = 7.580; 95% CI 1.852–31.029; P = 0.005). However, according to the conventional OSA classification based on AHI severity, there was no significant difference in disease-specific mortality (Log rank, P value = 0.331; Fig. 2b). Moreover, Cox proportional hazards model demonstrated no significant risk of mild to severe OSA compared to normal individuals with AHI less than 5/h after adjustment for age, BMI, sex, and comorbidities (Table 6). For all-cause mortality, both conventional OSA classification and clusters showed a significant difference. After adjustment for age, BMI, sex, and underlying medical condition, cluster 4 and severe OSA patients had significantly higher risk of all-cause mortality as compared to cluster 1 and normal individuals. However, the clusters showed no significant differences in mortalities due to other causes (Supplementary Figs. S2^,^S3; Tables S5^,^S6)

**Table 4:** Patient outcome according to clusters.

	Cluster 1	Cluster 2	Cluster 3	Cluster 4	Total	P value
**Cause of death**						
Cancer (%)	18 (0.8%)	3 (0.9%)	17 (1.4%)	4 (1.1%)	42 (1%)	0.300
Cardiovascular/cerebrovascular (%)	5 (0.2%)	4 (1.3%)	6 (0.5%)	4 (1.1%)	19 (0.5%)	0.011
Trauma (%)	10 (0.4%)	1 (0.3%)	6 (0.5%)	2 (0.5%)	19 (0.5%)	0.955
Suicide (%)	7 (0.3%)	1 (0.3%)	3 (0.3%)	1 (0.3%)	12 (0.3%)	0.996
Others (%)	14 (0.6%)	5 (1.6%)	15 (1.3%)	5 (1.4%)	39 (0.9%)	0.086
Total (%)	47 (2.0%)	13 (4.1%)	44 (3.7%)	15 (3.7%)	119 (2.8%)	0.004
**Underlying medical condition**						
Hypertension (%)	388 (15.1%)	92 (26.4%)	305 (23.9%)	112 (27.3%)	898 (19.5%)	< 0.001
Diabetes (%)	189 (7.4%)	43 (9.5%)	165 (12.9%)	55 (13.4%)	452 (9.8%)	< 0.001
Cardiovascular/cerebrovascular (%)	145 (5.7%)	28 (8.0%)	115 (9.0%)	28 (6.8%)	316 (6.9%)	< 0.001
Total cholesterol (mg/dL)	184.2 ± 30.4	180.8 ± 30.8	183.4 ± 30.7	187.9 ± 34.5	184.0 ± 30.9	< 0.001
Number of checked patients (%)	1806 (70.5%)	279 (79.3%)	934 (73.1%)	280 (68.3%)	3,299 (71.1%)	
SE	0.71	1.85	1.00	2.06	0.54	
Range	77–348	95–306	100–306	111–303	77–348	
HDL (mg/dL)	52.5 ± 11.8	50.8 ± 11.8	49.7 ± 10.7	46.3 ± 9.1	51.1 ± 11.5	< 0.001
Number of checked patients (%)	1,303 (50.8%)	234 (66.5%)	656 (51.3%)	188 (45.9%)	2,381 (51.7%)	
SE	0.33	0.77	0.42	0.67		
Range	23–104	22–101	22–89	12–77	12–104	
Smoking (%)	37.2 (384/1,033)	38.6 (66/171)	44.8 (265/592)	61.0 (111/182)	41.8 (826/1978)	< 0.001

**Table 5:** Cox hazard ratio and adjusted Cox hazard ratio for cardiovascular/cerebrovascular disease related mortality of OSA clusters.

	HR	95% CI	P value
Cluster 1	Reference		
Cluster 2	6.460	1.734–24.073	0.005
Cluster 3	2.281	0.696–7.476	0.173
Cluster 4	4.844	1.300–18.047	0.019

	Adjusted HR	95% CI	P value
Age	1.084	1.037–1.134	< 0.001
BMI	0.896	0.770–1.044	0.159
Sex	1.028	0.377–2.804	0.956
HTN	0.688	0.229–2.071	0.506
DM	1.352	0.413–4.424	0.618
CVD	3.708	1.307–10.519	0.014
Cluster 1	Reference		
Cluster 2	3.127	0.819–11.942	0.095
Cluster 3	1.862	0.541–6.403	0.324
Cluster 4	7.580	1.852–31.029	0.005

*BMI* Body mass index, *HR* hazard ratio, *CVD* cardio/cerebrovascular disease, *DM* Diabetes mellitus, *HTN* hypertension.

**Table 6:** Cox hazard ratio and adjusted Cox hazard ratio for cardiovascular/cerebrovascular disease related mortality of OSA severity classification.

	Adjusted HR	95% CI	P value
Age	1.085	1.040–1.132	< 0.001
BMI	0.913	0.777–1.072	0.265
Sex	0.964	0.353–2.631	0.943
HTN	0.690	0.229–2.082	0.511
DM	1.331	0.402–4.402	0.640
CVD	3.719	1.304–10.608	0.014
**OSA severity**			
Normal (AHI < 5)	Reference		
Mild (5 ≤ AHI < 15	0.437	0.078–2.432	0.344
Moderate (15 ≤ AHI < 30)	0.816	0.194–3.431	0.782
Severe (AHI ≥ 30)	1.729	0.479–6.247	0.403

*AHI* Apnea–hypopnea index, *HR* hazard ratio, *CVD* cardio/cerebrovascular disease, *DM* diabetes mellitus, *HTN* hypertension.

## Discussion

The current classification of OSA is based on AHI severity only, since each OSA severity is known to have a different outcome as shown in a longitudinal cohort study^4^. The goal of this study was to identify multiple OSA phenotypes based on various PSG features and to assess whether these PSG features were distinct from each other. It is prudent to understand the meaning of PSG features, including AHI, to identify and understand the pathophysiology of OSA. Numerous studies have reported on the importance of other PSG parameters besides AHI^5–8^,however, these studies mainly focused on specific PSG parameters. There is a paucity of studies that have simultaneously analyzed and compared all PSG parameters.

All-cause mortality was significantly different for both conventional classification of OSA and cluster-based classification. The adjusted HR for all-cause mortality in severe OSA group was significantly higher compared to normal individuals. However, mortality differed significantly only among the clusters when cardiovascular or cerebrovascular disease were considered in the analysis. After adjustment for age, sex, BMI, and underlying comorbidities, cluster 4 had significantly higher risk of mortality due to cardiovascular or cerebrovascular diseases as compared to cluster 1, while patients with severe OSA did not have a significantly higher risk compared to normal individuals. The current result contradicts our previous report, which stated that patients with severe OSA had significantly higher risk of mortality due to cardiovascular and cerebrovascular diseases as compared to normal individuals. However, this contradiction seems to be related to the recent decreasing trends of mortality due to cardiovascular and cerebrovascular diseases in Korea^32,33^.

Although both cluster 1 and cluster 2 are mostly composed of patients with normal to mild OSA, they represent different clinical outcomes. Cluster 2 is significantly older, and is characterized by a high PLM index and low sleep efficiency. It is known that prevalence of PLM increases with age^34,35^ and it is also known to be associated with OSA, such that PLM syndrome (PLMS) is more common in patients with sleep-disordered breathing than in the general population^36^. One study demonstrated a decreased PLM after CPAP therapy in mild OSA, suggesting the development of PLM as one of the consequences of OSA^37^. However, PLM sometimes newly appears after the initiation of positive airway pressure therapy^38^ and therefore the direct causal relationship between PLMS and OSA is poorly understood

PLMs are followed by arousal-related nervous system events, which manifest as cortical activity, and heart rate increases significantly in the first 5 s after the PLM^39^. Previous studies have advocated the importance of PLM since events may be representative of decreased dopaminergic activity or increased activation of the autonomic nervous system, which may be related to increased vascular consequences^7,13,34,40^. In our study, cluster 2 had a significantly higher risk of disease-specific mortality compared to cluster 1. However, after adjustment for age, increased risk in cluster 2 was no longer significant. Therefore, whether poor outcomes in cluster 2 are due to age, or other clinical findings possibly related to characteristic PSG features needs to be further validated.

Another important cluster was cluster 4. This cluster was characterized by the most severe type of OSA with the highest AHI. Contrary to the other clusters, this cluster had the lowest proportion of hypopnea, ratio of REM AHI to NREM AHI, and ratio of supine AHI to lateral AHI. Although age, BMI, and ESS were not included in the clustering, this cluster had the highest ESS score, highest BMI, and the youngest age. Severe obesity and high ESS scores seem to be the cause and effect, respectively, of cluster 4. Increased sleep efficiency in this cluster may be the result of excessive daytime sleepiness^41^. In Korea, severe obesity is more prevalent in younger adults^42^, which may explain the relatively young age of cluster.

Patients in cluster 4 had the highest age-adjusted cardiovascular/cerebrovascular mortality. Similar trend was observed for patients with severe OSA (AHI ≥ 30), a statistical significance was lacking, suggesting that not all patients in severe OSA carry a similar cardiovascular/cerebrovascular risk. PSG measurements representing the cumulative exposure to hypoxemia during sleep (i.e. time of O_2_ saturation < 90%), or depth and duration of hypoxia induced by respiratory distress, are known to be strongly associated with cardiovascular mortality^43,44^. However these measurements are only moderately associated with AHI, and therefore may not be captured by frequency based metrics only. Time of O_2_ saturation < 90% and mean apnea hypopnea duration were highest in cluster 4 than in the other clusters suggesting that patients in cluster 4 are grouped by combinations of PSG measurements associated with adverse cardiovascular outcome. This seems to result in additional cardiovascular/cerebrovascular mortality risk in cluster 4.

Cluster 3, which had a mean AHI of 38.6 ± 13.65/h, had a higher level of fraction of hypopnea, ratio of REM AHI to NREM AHI, and ratio of supine AHI to lateral AHI compared with cluster 4. This cluster had lower mean apnea–hypopnea durations. The fraction of hypopnea, sleep stage dependency, position dependency, and duration of breathing disturbance are known to be related to other pathophysiological traits. For example, increased fraction of hypopnea may indicate decreased arousal threshold, while increased apnea–hypopnea duration with desaturation may indicate increased arousal thresholds^45^. The ratio of REM AHI to NREM AHI may also indicate alterations in muscle responsiveness with higher values suggesting higher muscle responsiveness^45,46^. Although most of the patients in clusters 3 and 4 belonged to the same category of severe OSA, the PSG characteristics and clinical outcomes were different between the two clusters, which may suggest different underlying pathophysiologies.

As the mean BMI was the highest in cluster 4, severe obesity may have resulted in different PSG characteristics and disease outcomes in this cluster. The AHI, ratio of REM AHI to NREM AHI, ratio of supine AHI to lateral AHI, and severity of nocturnal hypoexmia are known to be correlated to BMI^30,31^. However, severe obesity cannot explain all the observed features of cluster 4. In this cluster, 16.6% (68/410) of the patients were non-obese (BMI < 25 kg/m^2^). Although the mean AHI and mean ODI scores were higher in the obese group, mean AHI in the non-obese group was still high (62.85 events/h), and there was no significant difference in the ratio of REM AHI to NREM AHI and ratio of supine AHI to lateral AHI between the obese and non-obese groups.

Cluster analysis with variables limited to breathing disturbance and desaturation (such as AHI, ODI, time of O_2_ saturation < 90%, and lowest O_2_ saturation) may be a simpler approach compared to our study which used 29 PSG variable from 5 different domains. One recent study demonstrated that oximetric parameters were able to describe a different phenotype with a high risk of mortality among patients with moderate to severe OSA^47^. However, our study revealed how other PSG features were grouped together so that we could characterize each cluster from many other perspective. In either way, these analytic methods may ultimately help us to understand the pathophysiology of OSA by phenotyping patients with PSG features beyond AHI.

The current study has several limitations. First, no adjustment was made for patient treatments. CPAP therapy is the gold standard procedure to treat OSA; however, the long-term, definitive effects of CPAP to prevent adverse cardiac events, stroke, or death are still controversial^48–50^. Some of the patients in our study population may have been using CPAP therapy. However, until 2018, CPAP therapy was not covered by the insurance system in Korea. Therefore, CPAP treatment was used as an off-label therapy and no systematic data are available on the use of CPAP therapy. Presently, CPAP treatment is covered by insurance, therefore, future prospective studies to validate the effect of CPAP treatment in disease prevention are necessary. Our clustering algorithm may improve the efficacy of CPAP therapy for certain patient groups. Second, there were no adjustments for the treatment of underlying comorbid diseases. Although serum cholesterol, smoking history were collected, these were not adjusted in survival analysis. These are the limitations that originated from the retrospective study design. A relatively short follow up period and therefore a low event rate is another limitation. A longer follow up period or perhaps prospective multicenter studies, with more detailed clinical information may overcome these limitations. In addition, this is a single center study mainly composed by a Korean population, therefore, generalization should be avoided as ethnic factors such as diet, craniofacial morphological differences, obesity, and prevalence of comorbidities are known to be different from Western countries^51,52^. Finally, K-means clustering may not be the optimal clustering algorithm for the current dataset. Theoretically, K-means clustering assumes that all clusters lie within a sphere with the same radius. When this equal-radius, spherical assumption is violated, as in the case of elliptically distributed data, K-means clustering can behave non-intuitively. Moreover, the number of K of groupings is fixed, and therefore, groupings would be easily violated by a small number of outliers^53^. Therefore, our results need to be further validated by other clustering algorithm.

## Conclusion

This study identified four distinct clusters of patients with OSA solely based on the various PSG features. There was a significant difference in disease outcome among the clusters, and such a difference could not be found in the standard classification of OSA based on AHI severity scores. Distinct characteristics among clusters imply different underlying pathophysiologies. Based on these phenotypes, further understanding and personalized treatment of OSA can be made. Our findings suggest that physicians should consider the AHI score along with other PSG parameters in their assessment of patients with OSA.

## Supplementary information

Supplementary information

## Data Availability

The data cannot be publicized for legal reasons in Korea.
