# Supplementary material for: Polysomnographic phenotyping of obstructive sleep apnea and its implications in mortality in Korea
Source: Sci Rep. 2020 Aug 6;10:13207. doi: 10.1038/s41598-020-70039-5 (PMC7411028; doi:10.1038/s41598-020-70039-5)
Supplement: Supplementary file 1 — Supplementary information [file 41598_2020_70039_MOESM1_ESM.docx]

**Supplementary data**

**Polysomnographic phenotyping of obstructive sleep apnea and its implications in mortality in Korea**

Jeong-Whun Kim, Tae-Bin Won, Chae-Seo Rhee, Young Mi Park, In-Young Yoon, Sung-Woo Cho.

**Table S1. Reported polysomnographic variables.** In Polysomnography report, 63 variables are reported. These variables are categorized as below. From these variables, 29 variables (*) were selected and were used for analysis.

| **Domain** | **Parameters** |
| --- | --- |
| **Anthropometric variables** | Height |
|  | Weight |
|  | Body Mass Index |
|  | Neck Circumference |
|  | Waist Circumference |
|  | Hip Circumference |
| **Questionnaire** | Pittsburg Sleep Quality Index |
|  | Epworth Sleepiness Scale |
| **Oropharynx Anatomy** | Tonsil Grade |
|  | Palatal Grade |
|  | Friedman Stage |
| **Sleep architecture** | Time In Bed (recorded time) |
|  | **Sleep Latency*** |
|  | Total Sleep Time |
|  | Wake After Sleep Onset |
|  | **Sleep Efficiency*** |
|  | Number Of Awakening |
|  | **REM Latency*** |
|  | **Stage 1 NREM*** |
|  | **Stage 2 NREM*** |
|  | **Stage 3 NREM*** |
|  | **REM%*** |
| **Breathing disturbance** | **Apnea Hypopnea Index (AHI) *** |
|  | **Apnea Index*** |
|  | **Obstructive Apnea Index*** |
|  | Central Apnea Index |
|  | **Mixed Apnea Index*** |
|  | **Hypopnea Index*** |
|  | Hypopnea Index With Desaturation |
|  | Hypopnea Index Without Desaturation |
|  | **AHI During REM Sleep*** |
|  | **AHI During NREM Sleep*** |
|  | Percent Sleep Time In REM |
|  | Percent Sleep Time In NREM |
|  | **AHI In Supine Position*** |
|  | **Percent Sleep Time In Supine Position*** |
|  | AHI In Left Lateral Position |
|  | Percent Sleep Time In Left Lateral Position |
|  | AHI In Right Lateral Position |
|  | Percent Sleep Time In Right Lateral Position % |
|  | Longest Apnea Duration |
|  | **Mean Apnea Duration*** |
|  | **Mean Hypopnea Duration*** |
|  | **Mean Total Apnea And Hypopnea Duration*** |
| **Desaturation** | Waking Oxygen Saturation |
|  | **Average Oxygen Saturation*** |
|  | **Lowest Oxygen Saturation*** |
|  | **Time of Saturation < 90%*** |
|  | Time of Saturation < 80% |
|  | Time of Saturation < 70% |
|  | Time of Saturation < 60% |
|  | **Oxygen Desaturation Index*** |
| **Snoring** | Snoring Time |
|  | Number of Snoring Episodes |
|  | Average Snoring Episode Duration |
|  | Longest Snoring Episode |
| **Limb movement** | **Limb Movement*** |
|  | **Periodic Limb Movement Index*** |
| **Arousal indices** | **Limb Movement With Arousal*** |
|  | **Respiratory Arousal*** |
|  | **Periodic Limb Movement Arousal*** |
|  | **Spontaneous Arousal*** |

# Table S2. Results from principal component analysis. 8 components were selected. As these components retained 75% of the total variance. After application of varimax rotation to maximize item variance and simplify interpretability, scores of given principal components representing PSG features were acquired for each subject.

| **Component** | **Eigenvalue** | **Proportion** | **Cumulative variance (%)** |
| --- | --- | --- | --- |
| **1** | **10.04** | **34.62** | **34.62** |
| **2** | **2.93** | **10.10** | **44.72** |
| **3** | **2.14** | **7.40** | **52.11** |
| **4** | **1.83** | **6.33** | **58.44** |
| **5** | **1.47** | **5.08** | **63.52** |
| **6** | **1.29** | **4.44** | **67.96** |
| **7** | **1.15** | **3.98** | **71.94** |
| **8** | **1.07** | **3.70** | **75.64** |
| **9** | **0.90** | **3.11** | **78.74** |
| **10** | **0.87** | **2.99** | **81.74** |
| **11** | **0.85** | **2.94** | **84.68** |
| **12** | **0.76** | **2.63** | **87.31** |
| **13** | **0.71** | **2.44** | **89.75** |

**Figure S1. Optimal number of cluster determined by the Gap static method.** fviz_nbclust() function [in factoextra R package] was used with 500 bootstraps. The plot shows the number of clusters (**k**) with standard errors drawn with vertical segments and the optimal value of **k** marked with a vertical dashed blue line.

**
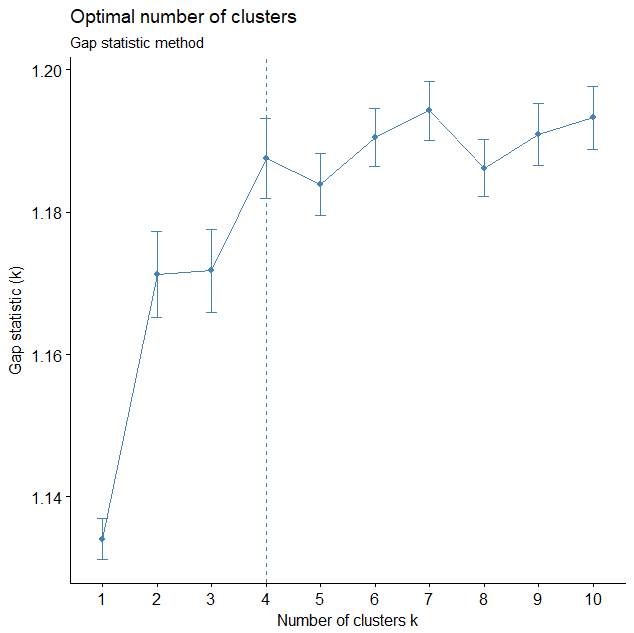
**

**Table S3 Cluster validation.** Cluster validation was done with Dunn index and average silhouette width a number of luster being tested from 3 to 5. For both indices, large values indicate good clustering.

| **Number of Cluster**  **Validation index** | **3** | **4** | **5** |
| --- | --- | --- | --- |
| **Dun index** | 0.0221 | 0.0218 | 0.0183 |
| **Average silhouette width** | 0.1969 | 0.2152 | 0.1501 |

**Table S4. The polysomnographic features of obese and non-obese patients in cluster 4**

| Parameters | Non Obese  N=68 | Obese  N=342 | P-value |
| --- | --- | --- | --- |
| Age (yr) | 52.81±13.02 | 47.26±10.99 | <0.001 |
| BMI (Kg/m^2^) | 23.48±1.39 | 30.00±3.76 | <0.001 |
| AHI(/hr) | 62.80±13.92 | 71.02±14.29 | <0.001 |
| Obstructive apnea index (/hr) | 45.58±15.42 | 52.60±18.64 | 0.004 |
| Hypopnea index (/hr) | 7.90±9.29 | 10.35±9.14 | 0.045 |
| Mixed apnea index (/hr) | 8.48±10.80 | 7.31±10.57 | 0.411 |
| Sleep Efficiency (%) | 80.66±10.90 | 85.15±9.35 | <0.001 |
| Stage1 NREM (% of TST) | 25.53±11.23 | 24.07±11.60 | 0.344 |
| Stage3 NREM (% of TST) | 2.63±4.69 | 3.36±4.37 | 0.213 |
| REM (%) | 13.60±6.93 | 15.21±6.10 | 0.053 |
| Sleep latency(min) | 13.52±16.82 | 11.21±16.09 | 0.285 |
| REM latency (min) | 145.85±85.34 | 132.52±74.73 | 0.191 |
| AHI REM/NREM | 0.90±0.38 | 0.85±0.24 | 0.143 |
| AHI Supine/Lateral | 2.97±4.90 | 2.84±8.15 | 0.920 |
| Time of O2 saturation < 90% (% of TST) | 21.51±16.51 | 32.47±19.17 | <0.001 |
| ODI (/hr) | 57.36±13.40 | 66.62±14.41 | <0.001 |
| Mean apnea duration (sec) | 34.48±8.30 | 30.11±7.26 | <0.001 |
| Mean apnea hypopnea duration (sec) | 34.39±7.81 | 29.83±7.18 | <0.001 |
| PLM (/hr) | 1.09±4.13 | 0.90±4.04 | 0.722 |
| Respiratory Arousal (/hr) | 53.73±12.37 | 61.33±14.82 | <0.001 |
| PLM arousal (/hr) | 0.12±0.56 | 0.09±0.58 | 0.709 |
| Spontaneous arousal (/hr) | 0.80±1.22 | 0.80±1.22 | 0.906 |

Abbreviations : AHI-Apnea-hypopnea index, NREM- non rapid eye movement, ODI – oxygen desaturation index, PLM – periodic limb movement, REM – rapid eye movement. TST –total sleep time. * by one way independent t-test.

**Table S5. Cox hazard ratio (HR) and adjusted Cox hazard ratio for all-cause mortality of OSA clusters**

|  | **HR** | **95% CI** | **P-value** |
| --- | --- | --- | --- |
| Cluster 1 | Reference | | |
| Cluster 2 | 2.294 | 1.241-4.241 | .008 |
| Cluster 3 | 1.756 | 1.164-2.650 | .007 |
| Cluster 4 | 1.899 | 1.062-3.396 | .031 |
|  | **Adjusted HR** | **95% CI** | **P-value** |
| Age | 1.069 | 1.051-1.086 | <0.001 |
| BMI | .0.964 | 0.908-1.023 | 0.223 |
| Sex | 1.107 | 0.739-1.657 | 0.623 |
| CVD | 1.235 | 0.708-2.155 | 0.457 |
| DM | 1.139 | 0.674-1.927 | 0.627 |
| HTN | 0.840 | 0.534-1.323 | 0.452 |
| Cluster 1 | Reference | | |
| Cluster 2 | 1.196 | 0.639-2.239 | 0.615 |
| Cluster 3 | 1.506 | 0.977-2.320 | 0.063 |
| Cluster 4 | 2.630 | 1.409-4.908 | 0.002 |

Abbreviations: BMI- Body mass index, HR-Hazard ratio, CVD-cardio/cerebrovascular disease, DM- Diabetes mellitus, HTN-Hypertension

**Table S6. Cox hazard ratio (HR) and adjusted Cox hazard ratio for all cause mortality of OSA severity**

|  | **HR** | **95% CI** | **P-value** |
| --- | --- | --- | --- |
| Normal | Reference | | |
| Mild | 1.389 | 0.741-2.602 | 0.305 |
| Moderate | 1.687 | 0.916-3.108 | 0.093 |
| Severe | 2.198 | 1.249-3.867 | .006 |
|  | **Adjusted HR** | **95% CI** | **P-value** |
| Age | 1.067 | 1.050-1.084 | <0.001 |
| BMI | 0.964 | 0.908-1.024 | 0.234 |
| Sex | 1.132 | 0.908-1.024 | 0.551 |
| CVD | 1.286 | 0.738-2.242 | 0.375 |
| DM | 1.137 | 0.672-1.924 | 0.632 |
| HTN | 0.827 | 0.526-1.302 | 0.412 |
| Normal | Reference | | |
| Mild | 1.243 | 0.657-2.352 | 0.504 |
| Moderate | 1.511 | 0.801-2.850 | 0.202 |
| Severe | 2.228 | 1.214-4.090 | 0.010 |

Abbreviations: BMI- Body mass index, HR-Hazard ratio, CVD-cardio/cerebrovascular disease, DM- Diabetes mellitus, HTN-Hypertension

**Figure S2.** All-cause mortality for clusters and conventional OSA classification. Both clusters (a) and conventional OSA classification (b) showed a significant difference (log rank = 0.006 and 0.029, respectively).


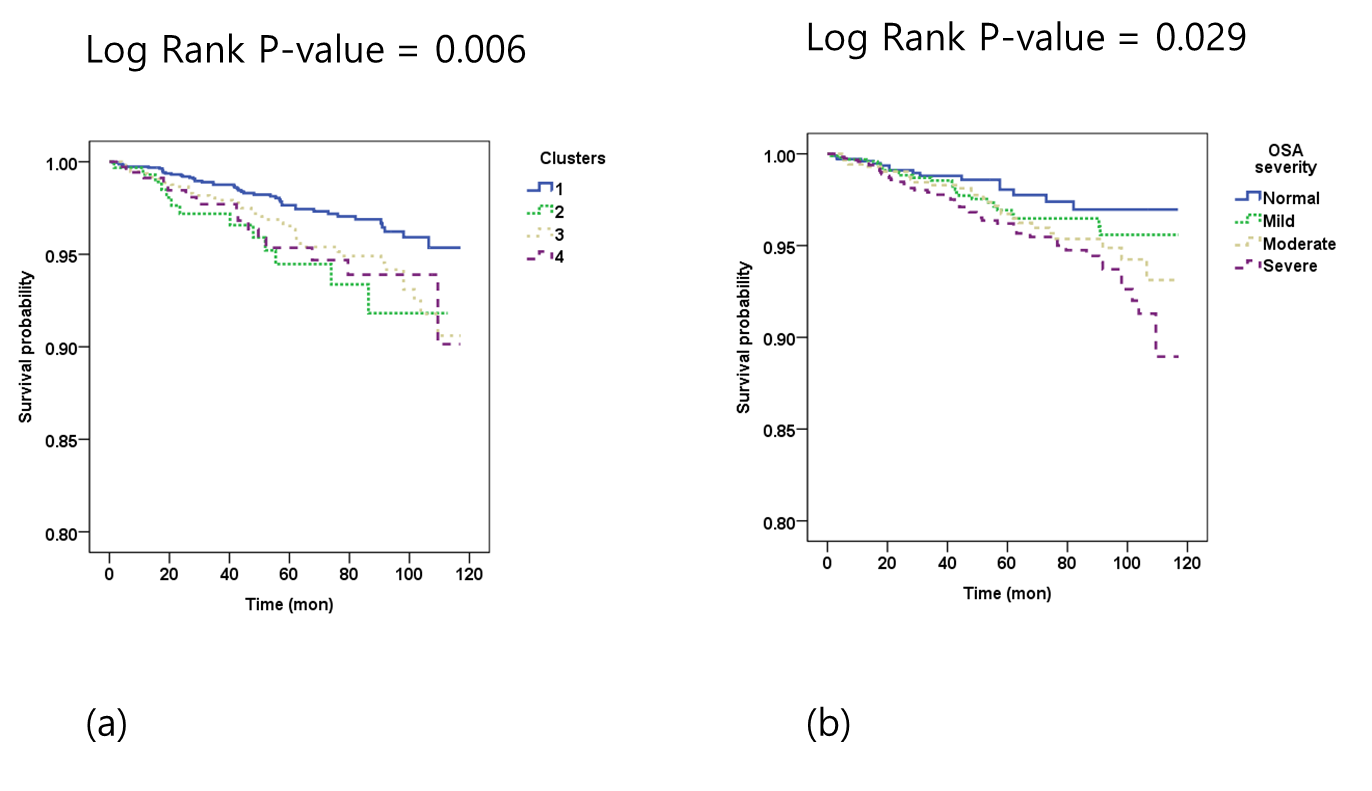


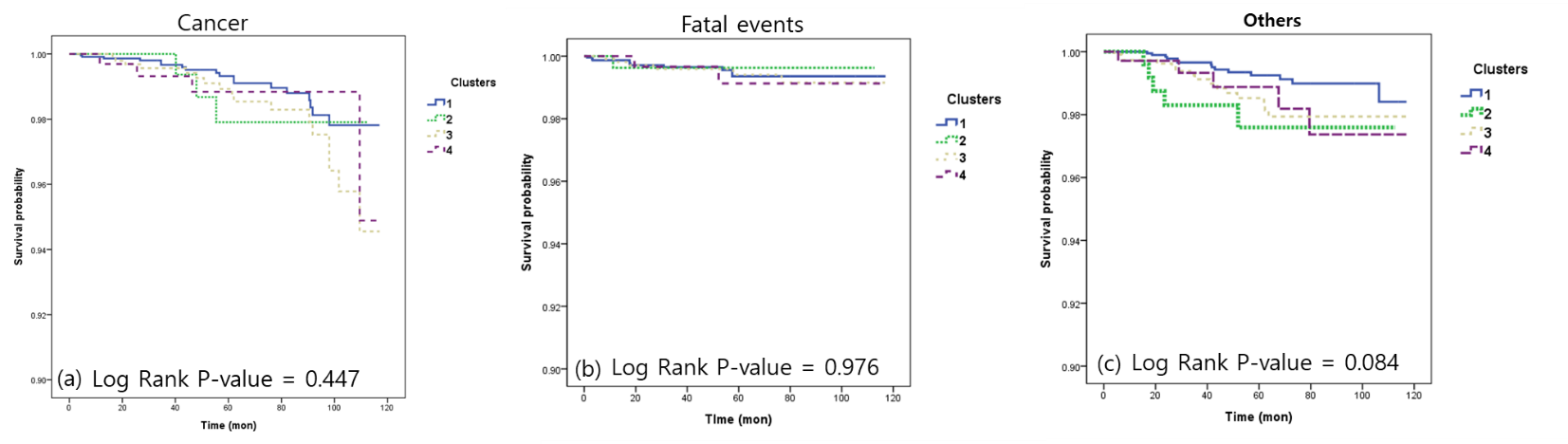
**Figure S3.** Disease specific mortality other than cardiovascular and cerebrovascular cause. Clusters did not show significant regarding cancer (a), fatal events (b), and other cause specific mortality (Log rank p-value = 0.447, 0.976, and 0.084, respectively)
